# Supplementary figures and images for: Feeling lucky? Prospective and retrospective cues for sensorimotor confidence
Source: PLoS Comput Biol. 2023 Jun 26;19(6):e1010740. doi: 10.1371/journal.pcbi.1010740 (PMC10348589; doi:10.1371/journal.pcbi.1010740)

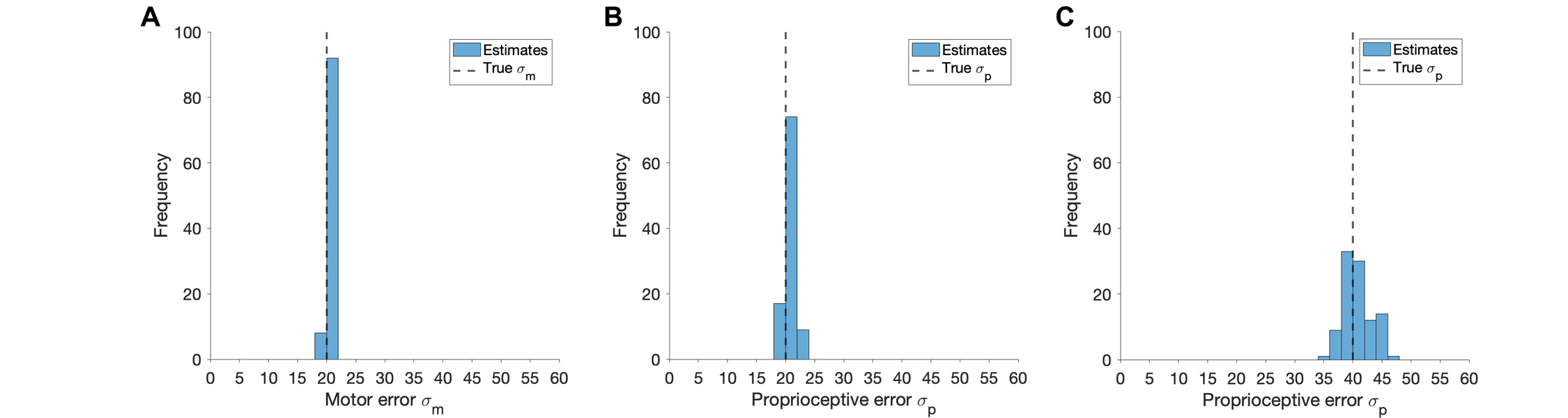

Supplement: S1 Fig — To test that a sufficient number of trials were collected the motor-awareness task, simulations were run for using various ground-truth parameter values. The low recovery error achieved with 300 trials was satisfactory. 100 iterations of 300 trials per simulated dataset. A) Estimates of motor error and ground-truth value. B) Estimates of proprioceptive error with a low sigma: very high accuracy. C) Estimates of proprioceptive error with a high sigma: less accurate but still close and unbiased. (TIF) [file pcbi.1010740.s001.tif]

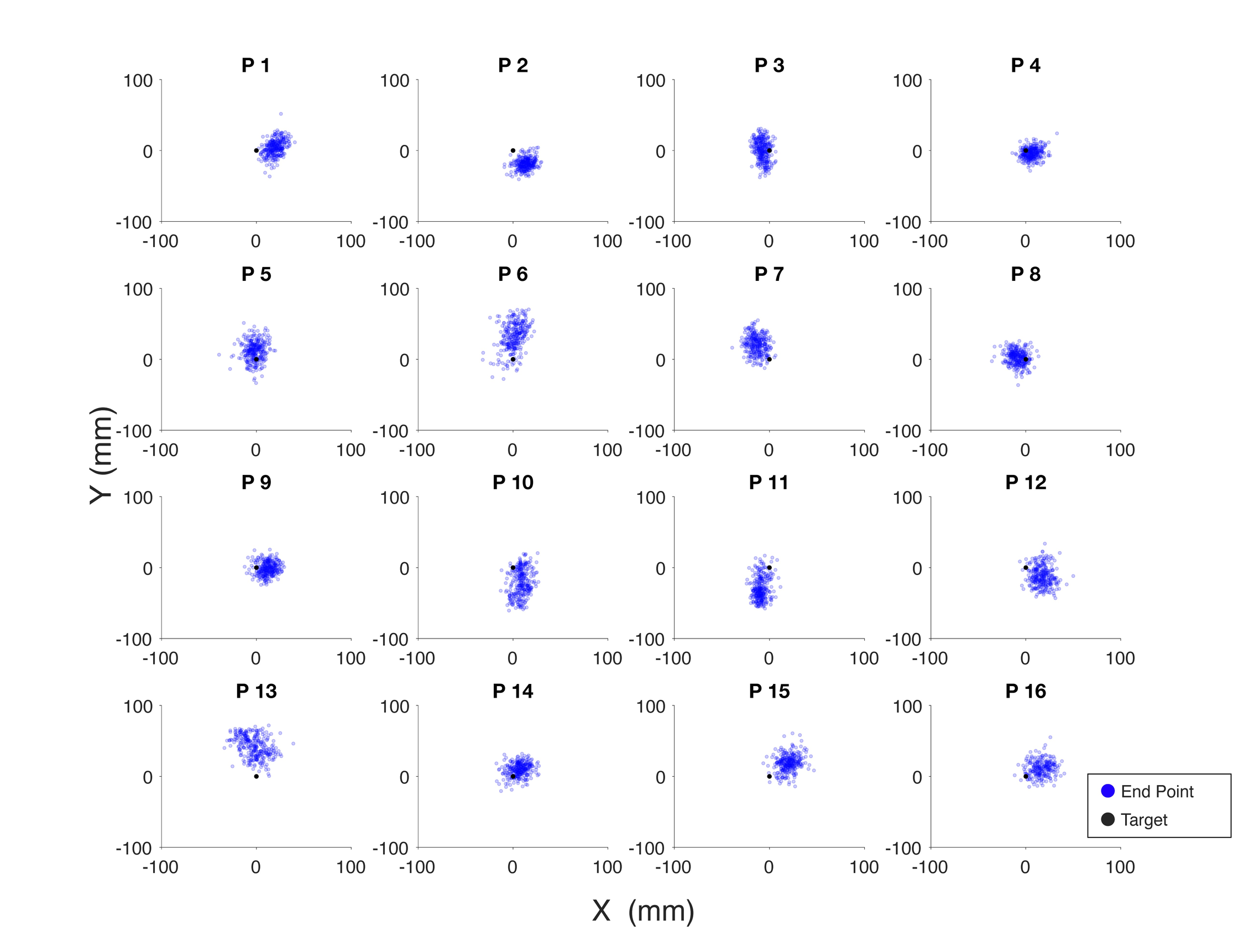

Supplement: S2 Fig — Endpoints (blue) shown around target location (black). For this task the target was in the same location on all trials. (TIF) [file pcbi.1010740.s002.tif]

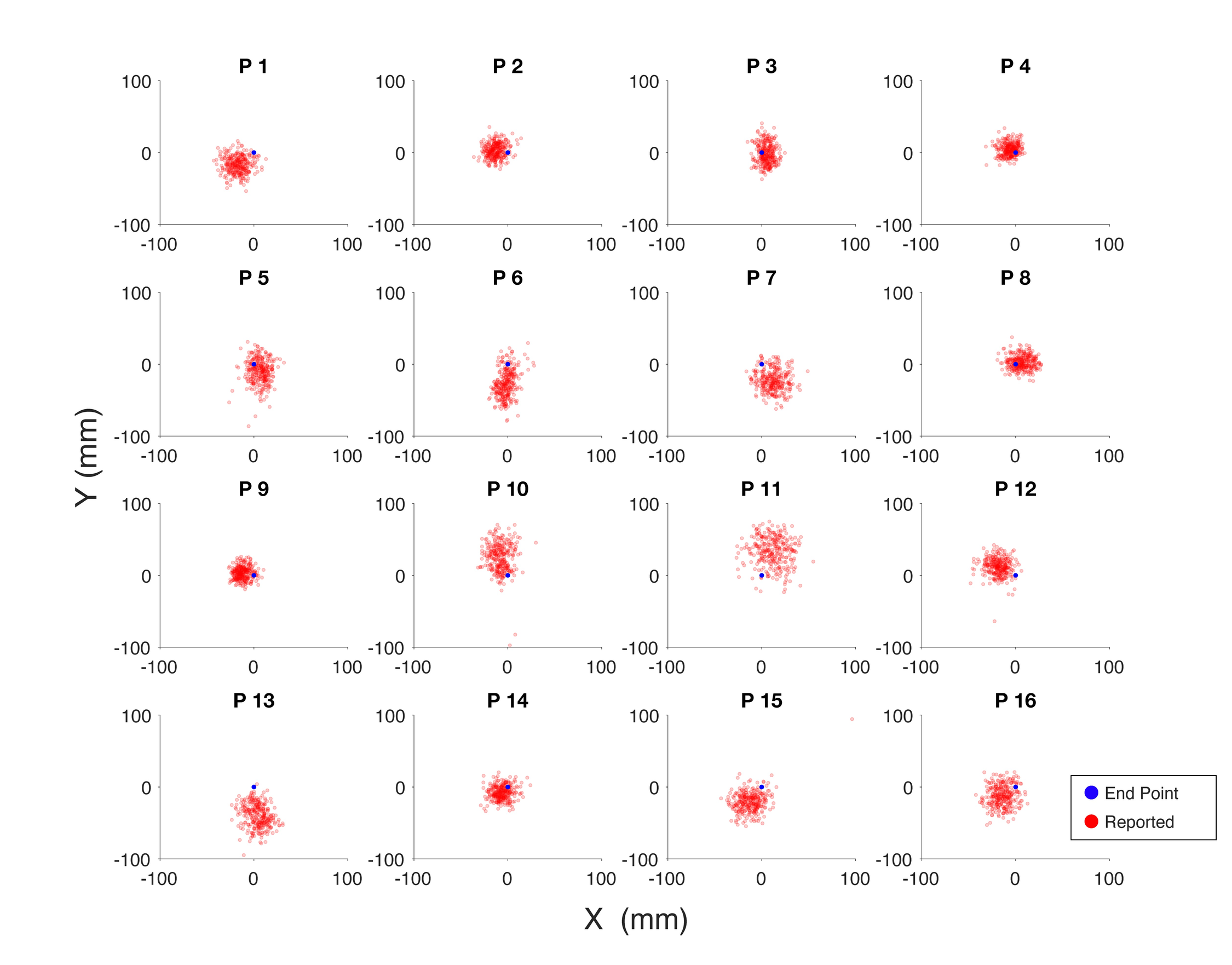

Supplement: S3 Fig — Reported endpoints (red) rotated and shifted so that the origin is the reach endpoint (blue). The y-axis is thus the radial direction (along the reach) and the x-axis is the tangential direction, orthogonal to the reach. The target was in the same location on all trials. However, the reach endpoints did not always hit the target spot on. When comparing these scatterplots to those in Fig. 14 you can see the influence of the prior (target location) on the reported endpoints as they show the opposite bias of the reaches. (TIF) [file pcbi.1010740.s003.tif]

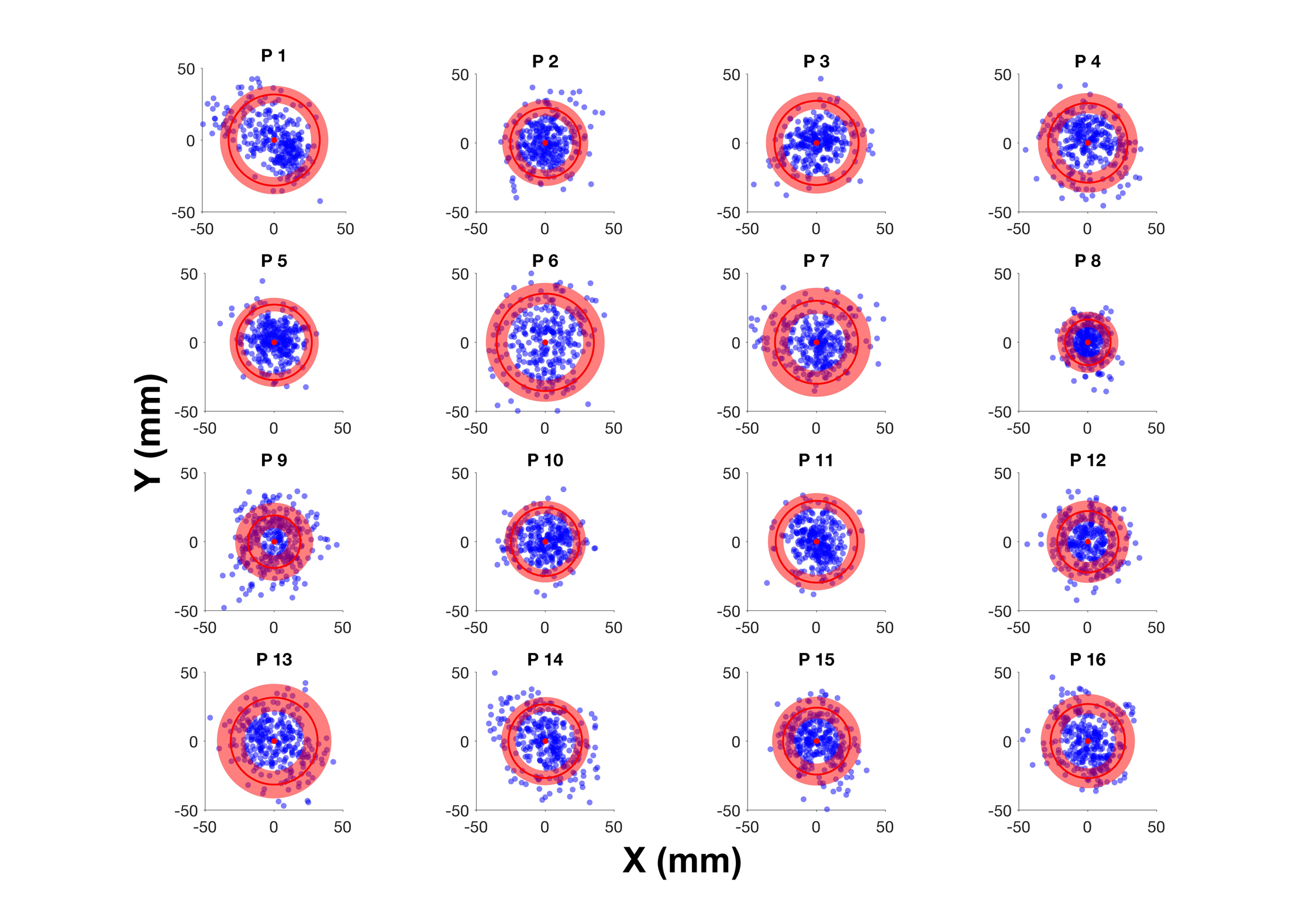

Supplement: S4 Fig — For the majority of our participants a Gaussian distribution was a good fit for the reach errors. Endpoint locations are rotated so that the y-axis is along the direction from start point to target and the x-axis is orthogonal to that direction. The red circle overlay shows the average confidence circle size in the center and ± SD for each participant as the transparent overlay. (TIF) [file pcbi.1010740.s004.tif]

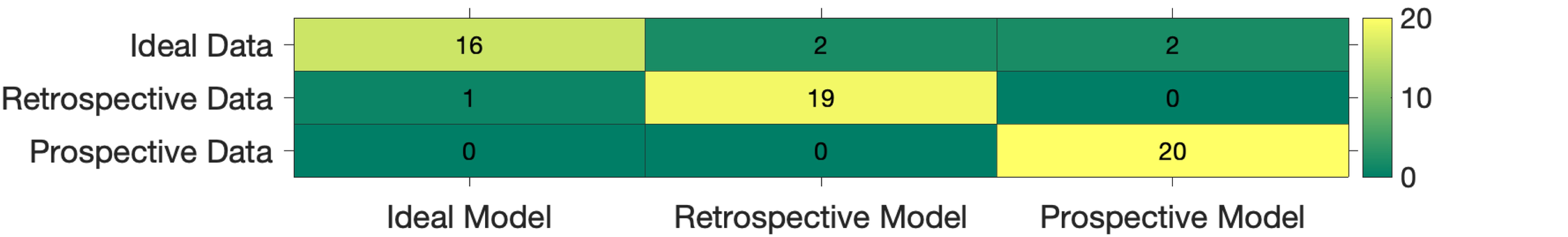

Supplement: S5 Fig — We performed a model recovery analysis to determine whether our experimental design would allow us to successfully recover the model that generated the data. We simulated data using 20 parameter sets. These 20 sets of {σm, σp, σs} were paired with each of our three models of confidence judgments, resulting in a total of 60 simulated datasets. Each simulation (i.e., combination of parameter set and confidence model) consisted of 300 simulated trials for Task 1 and 900 simulated trials for Task 2 (300 confidence reports and 600 reaches alone). The parameter values for each simulated data set were sampled from log-normal distributions with statistics based on our expectations for the experiment given participants’ performance (with mean and variance of σm: 20 mm and 25 mm2, σp: 35 mm and 400 mm2, σs: 7 mm and 25 mm2). Each model was fit to each simulated dataset by maximum likelihood and the best-fitting model was determined by BIC. For 56 of the 60 data sets, the correct underlying model was selected. This robust recovery indicates that the number of experimental trials is sufficient to identify the model that generated the data. The numbers in each square are the number of simulations that were best fit by a given model. (TIF) [file pcbi.1010740.s005.tif]

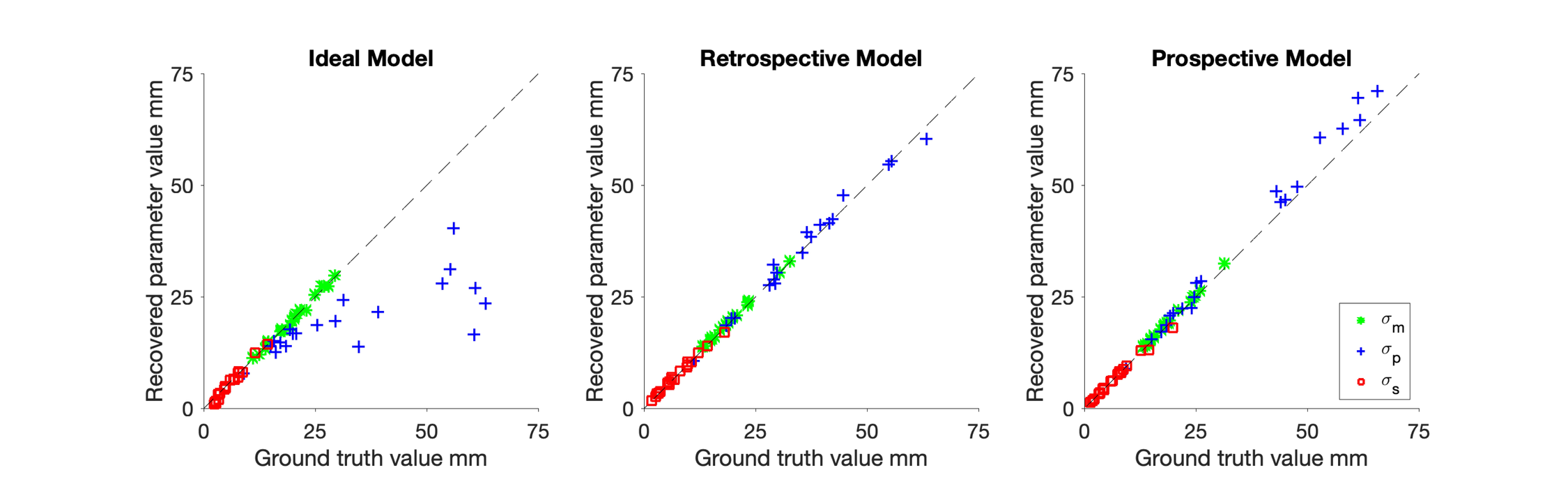

Supplement: S6 Fig — The best-fit parameters recovered by maximum likelihood for the ideal performance model under-predict the σp parameter when there is a model mismatch and ground truth σp is high (mean signed error in recovered parameter estimate as percentage of ground-truth value when σp > 30: 48%, SD: 16%) when the values were drawn from a log normal distribution with a mean of 40 and variance of 200 (S6A Fig). The degree of under-prediction was reduced when σp was drawn from a log normal distribution with a mean of 20 and variance of 200, the error in the parameter estimate as a percentage of the ground-truth value when σp < 30: 16.7%, SD: 9.1%. The other two parameters were recovered well regardless of the σp value, σm: 2.2%, SD:1.3%; σs: 15.7%, SD: 19.5%. Recovery of all three parameters was excellent for both the retrospective model (σm: 2.1%, SD:1.3%; σp,: 3.6%, SD: 3.8%; σs: 5.4%, SD: 4.4%) and the prospective model (σm: 2.8%, SD:1.3%; σp: 6.5%, SD: 4.3%; σs: 3.1%, SD: 2.7%). Data generated using the ideal model (green), the retrospective model (blue) and the prospective model (red). Parameters are indicated by the shape of the data point: motor noise (star), proprioceptive noise (plus) and setting noise (square). A) Parameter recovery for the ideal model for data generated using the ideal model (green), the retrospective model (blue) and the prospective model (red). B) Parameter recovery for the retrospective model for data generated using the ideal model (green), the retrospective model (blue) and the prospective model (red). C) Parameter recovery for the prospective model model for data generated using the ideal model (green), the retrospective model (blue) and the prospective model (red). (TIF) [file pcbi.1010740.s006.tif]

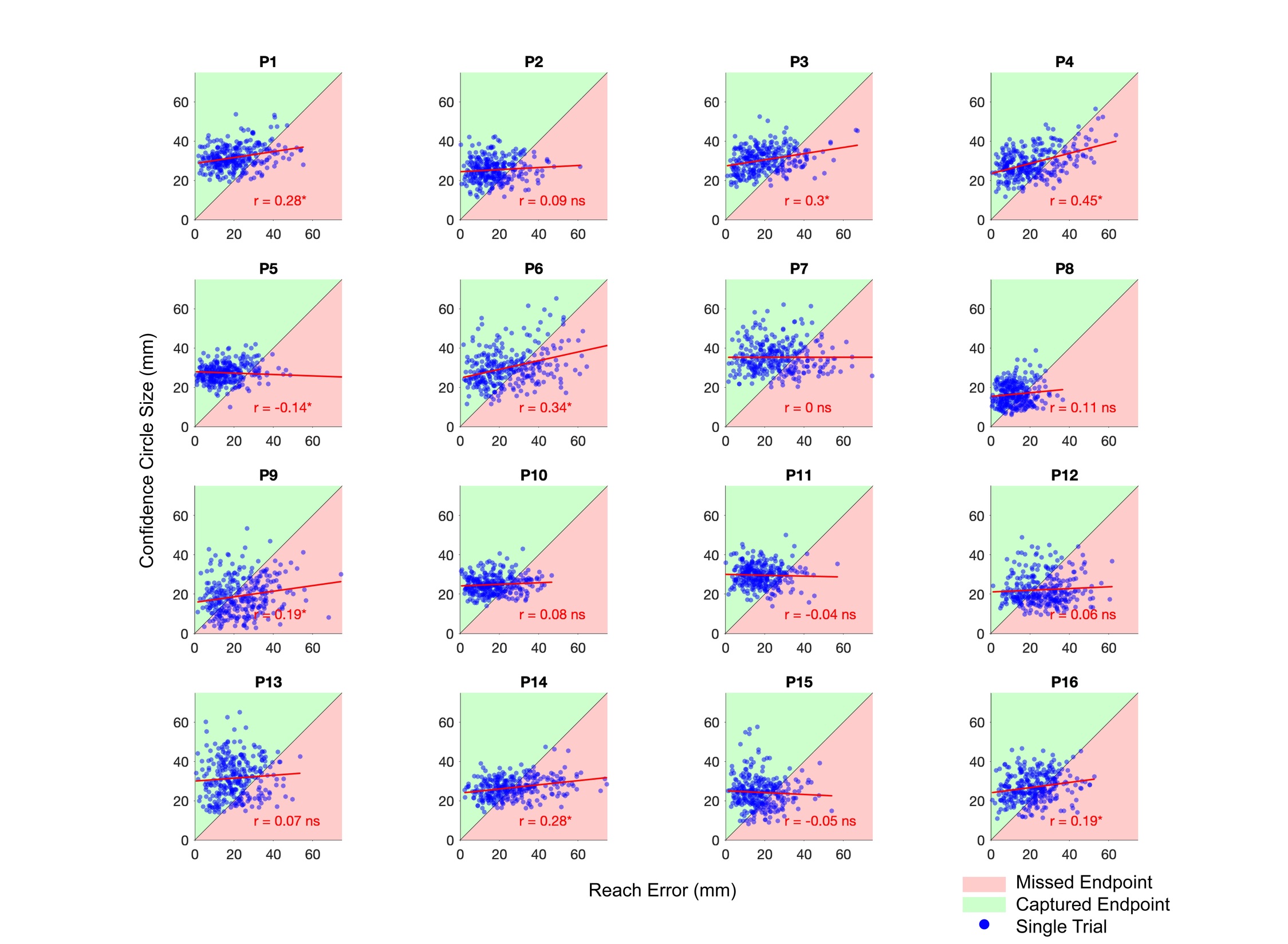

Supplement: S7 Fig — Correlations between Euclidian reach error distance and confidence circle size. Participants best fit by the Ideal performance model were more likely to have a significant correlation than those best fit by the Retrospective or Prospective models. Confidence circle size compared to reach error on each trial for all participants in Task 2. The green shaded area reflects judgements where the participant was able to earn points for enclosing their endpoint, and the red shaded area reflects judgements where the confidence circle was smaller than the distance from the endpoint to the target resulting in no points being awarded. *: Pearson’s r significant at the .05 level. (TIF) [file pcbi.1010740.s007.tif]

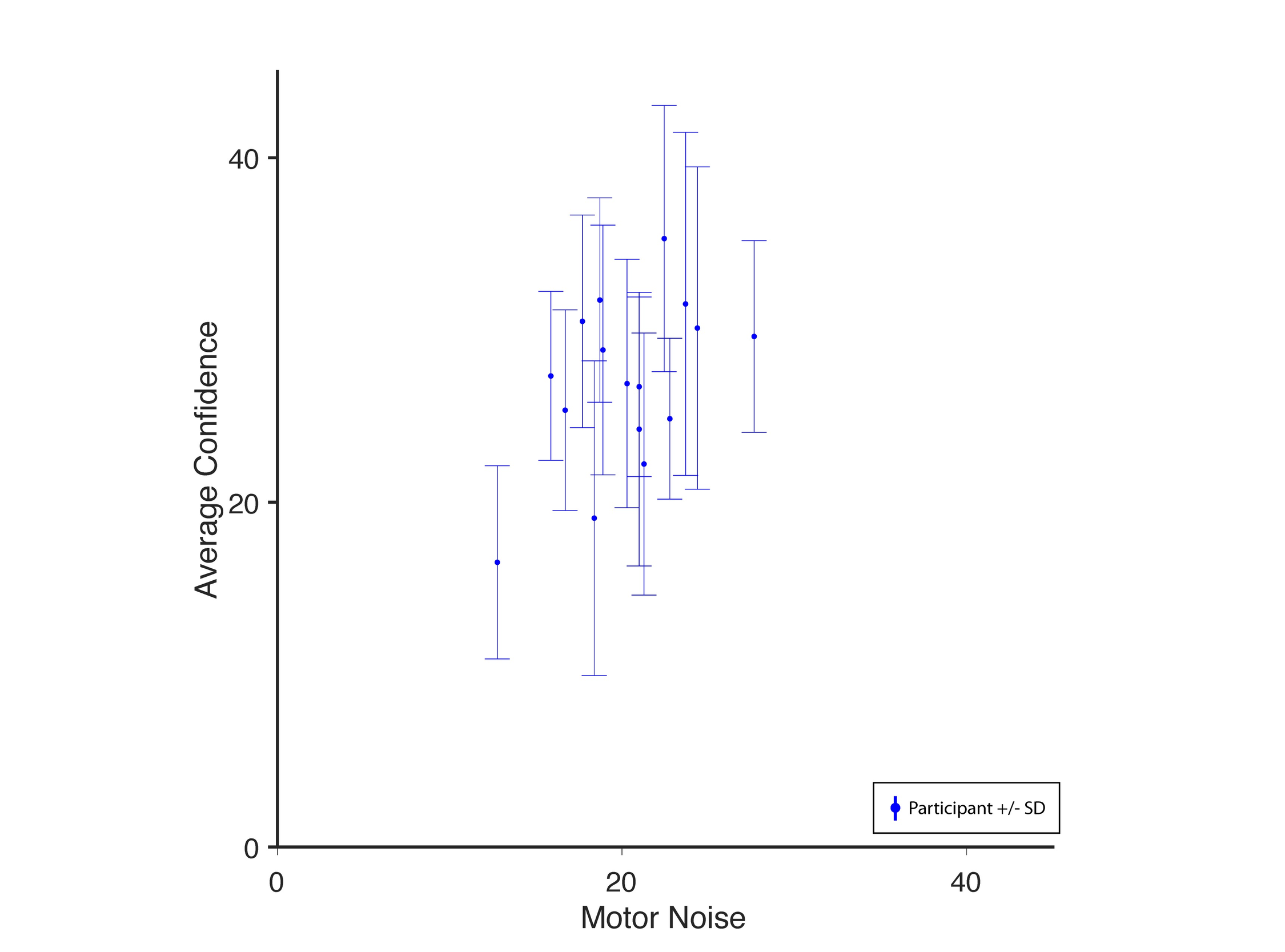

Supplement: S8 Fig — Average confidence is plotted +/- standard deviation for each participant compared to that participant’s motor noise. We can see a positive relationship between motor noise and confidence across participants with a Pearson’s r correlation of 0.5, significant at the .05 level. (TIF) [file pcbi.1010740.s008.tif]

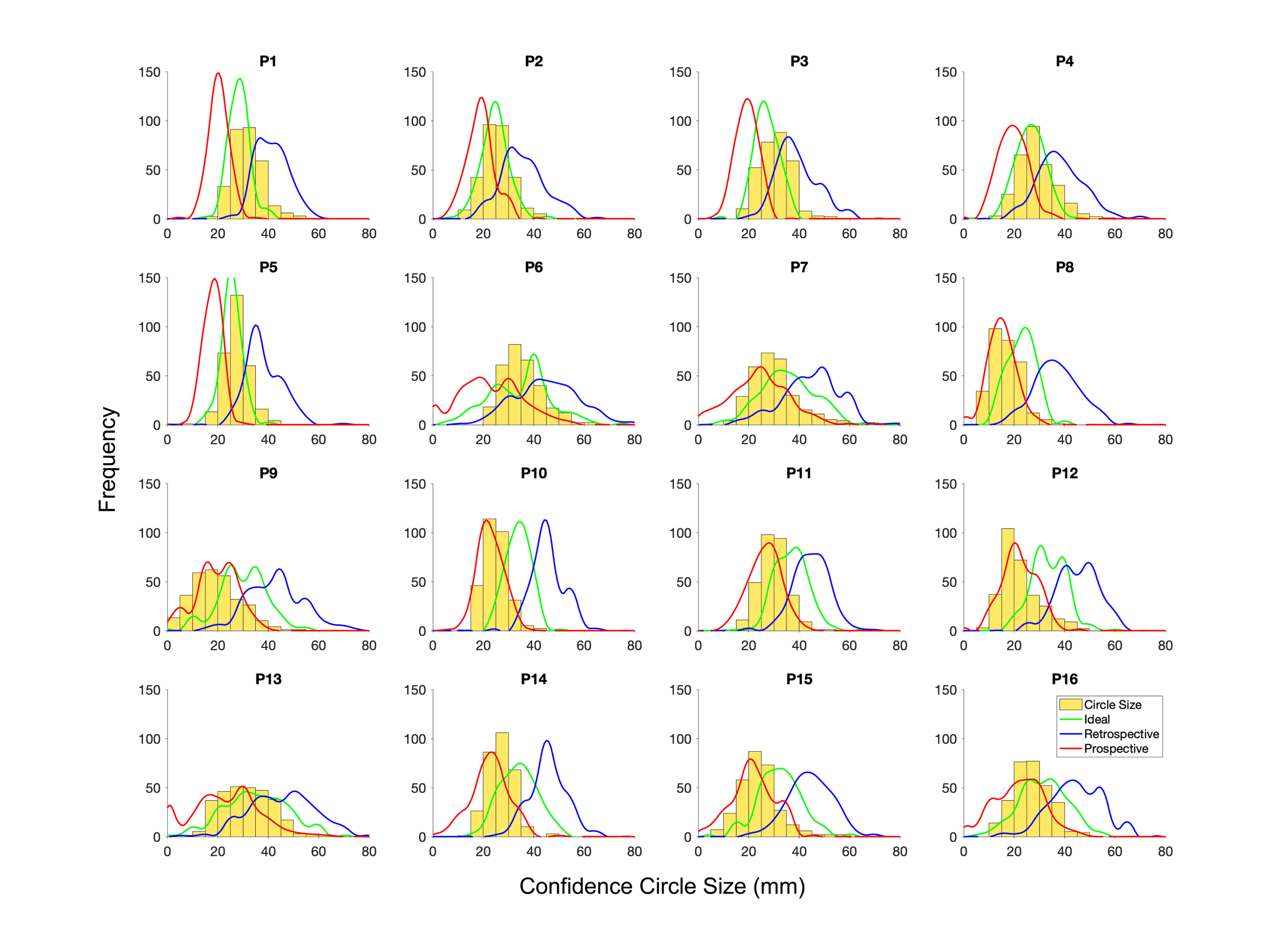

Supplement: S9 Fig — To compare our models to the data we simulated sets of maximum expected gain confidence circle sizes with each of the three models utilizing the target locations and endpoints from each participant’s data. The circle sizes simulated by the best-fitting model for each participant are the closest to the true circle sizes selected by that participant. Histograms showing selected circle size (yellow) for each participant. Data were simulated based on all three models using each participant’s parameters and reach endpoints, the distributions of circle sizes selected by the models are juxtaposed over the data. The ideal model (green), retrospective model (blue) and prospective model (red) are all shown. (TIF) [file pcbi.1010740.s009.tif]

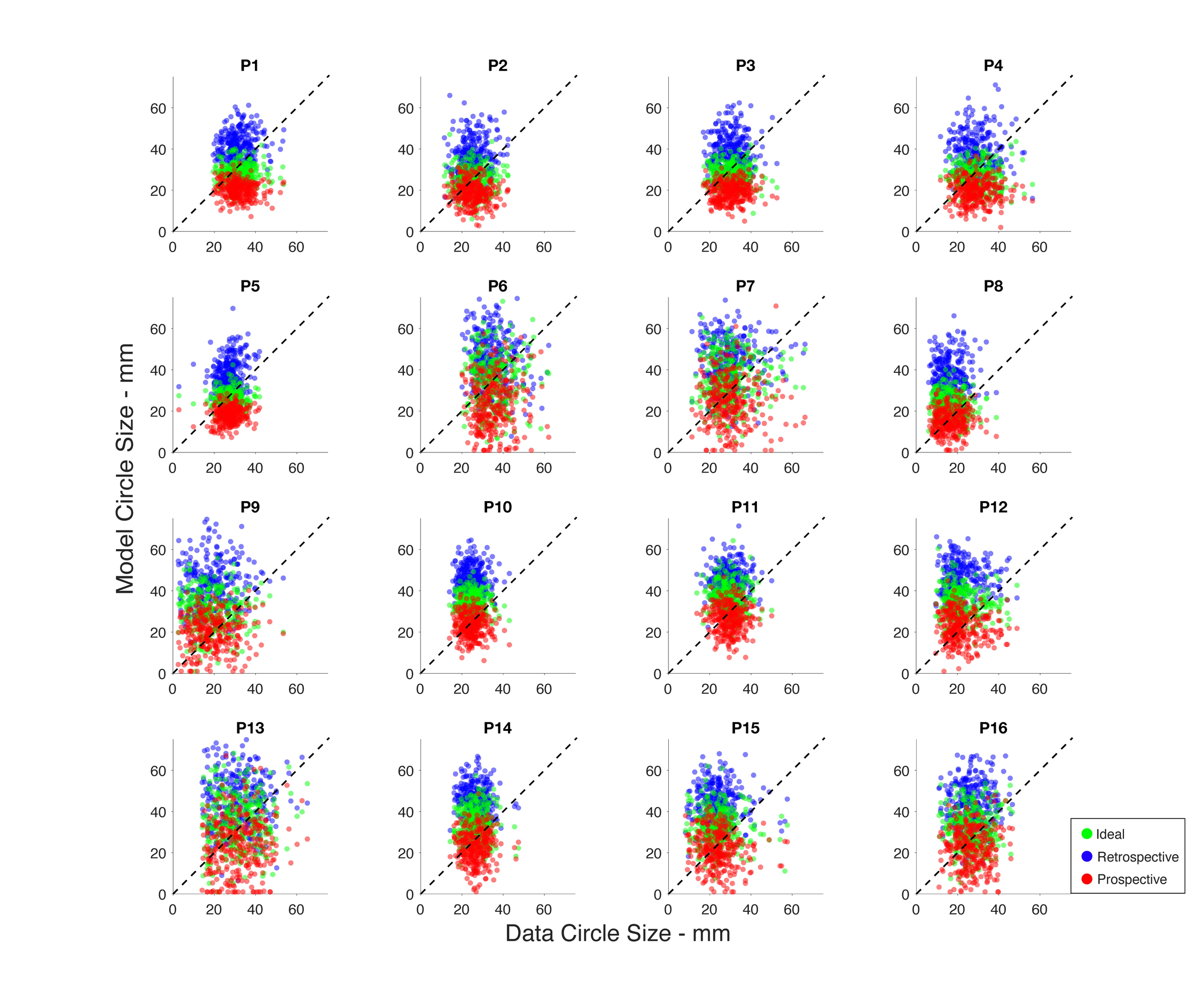

Supplement: S10 Fig — Scatter plots showing the circle sizes picked by each model compared to the actual circle sizes picked by the participant. The dashed line is the identity line. Participants 1–5 were best fit by the ideal model, and the circle sizes selected by that model are the closest match for those generated by the participant. The same can be seen for participants 7–16 as there is the greatest overlap between the prospective model circles and those chosen by the participant. Note that the small correlation in each cloud is due to the domination by real (for the data) and simulated (for the model) proprioceptive measurement noise. (TIF) [file pcbi.1010740.s010.tif]
