# Supplementary material for: Feeling lucky? Prospective and retrospective cues for sensorimotor confidence
Source: PLoS Comput Biol. 2023 Jun 26;19(6):e1010740. doi: 10.1371/journal.pcbi.1010740 (PMC10348589; doi:10.1371/journal.pcbi.1010740)
Supplement: Attachment — Submitted filename: Feeling Lucky_ Reviewer Response.pdf [file pcbi.1010740.s011.pdf]

Response to Reviewers - Feeling Lucky? PLoS Computational Biology Submission

Written by Marissa Fassold, Shannon Locke, Michael S Landy

Reviewer #1: Reviewer: Jonathan S. Tsay.

Summary: Fassold et al asked what cues are used to estimate endpoint hand position and to estimate the confidence associated with hitting the target. They found that participants behave like an ideal observer when proprioceptive signals are more reliable, that is, they incorporate both retrospective (i.e., a belief that the hand is at the target, informed by participants' movement history) and prospective cues (i.e., input from proprioceptive receptors on the current reach) in these estimates. However, when proprioceptive signals are less reliable, participants tend to rely only on retrospective cues. Together, these data shed light on the sensorimotor constraints underlying confidence judgments about hitting the target, taking us a step closer to understanding how our awareness of sensorimotor performance is determined.

Assessment: Fassfold et al tackled very important and timely questions. The methods and modeling are sound. The results are clear. Thank you!!

I have a few suggestions below that can hopefully strengthen the manuscript:

1) Confidence in hitting the target vs confidence in positional estimates. I understand that sensorimotor confidence refers to the confidence in successfully hitting the target, hence, that is why the circle is centered on the actual target location. It makes complete sense why prior information based on a history of motor errors (i.e., motor noise) is used here to make a judgment. I have no qualms about the underlying (and elegant) Bayesian principles outlined in the manuscript.

However, could the current task bias the results toward relying on prior information? That is, the participants are asked to return to the start position before making a confidence judgment, as opposed to holding the actual hand position in its original location when making a confidence judgment (e.g., (Ruttle et al., 2021; Tsay et al., 2020), but note that these are not confidence judgments but positional judgements). The latter simulates a real-life scenario in which one reaches forward to grab an object, misses rightward, detects a rightward error presumably using prospective proprioceptive cues in an online manner, and makes a corrective movement leftward. Together, it seems like moving the hand back to the start position may introduce potential forgetting or interference (i.e., proprioceptive cues from other locations), which may decrease the relative weight of proprioceptive cues on the confidence judgments. This would predict that the weight of proprioceptive cues may be used more if the task were designed differently, and potentially introduce alternative models (ideal observer + forgetting rate dependent on time; ideal observer + interference) for future tests.

We have added a comment in the discussion noting that the paradigm may have limited proprioception as participants didn't keep their hand in the endpoint location while reporting their confidence, which may possibly contribute to proprioception not being used by all participants, p. 38-39 paragraph 2

In addition, if the task were to estimate the confidence around the positional estimate of the hand after a movement (i.e., setting the confidence ring around the hand, rather than the target), would the authors expect participants to give greater weight to prospective proprioceptive cues rather than retrospective cues? I understand that this question may be outside the scope of the current study, but seems like a possible caveat to discuss since it may speak to how a little tweak in the question/task could potentially bias use of one cue over another.

I think it's important to distinguish here between having high confidence in your hand location, but knowing that you are nowhere near the target, and low confidence that you were successful at the goal of the reach having missed the target. We are explicitly asking participants to report how successful they were at the task, regardless of how confident they feel about their measurement of their true hand location. While both these lines of questioning are interesting and worth exploring, we define sensorimotor confidence as a rating dependent on the goal of the action (thus the target centered report), and motor awareness confidence as the rating dependent on the location of the end point. We've added a more detailed comment in the discussion regarding this distinction, p. 43 paragraph 1

With our center-out design, centering the confidence report circle on the endpoint location would shift the task to a report of the confidence in the memory of the target location and would not enlist use of proprioception if the endpoint location is provided by the experimenter (center of the circle).

2) What signals inform the development of participants' prior belief that their hand is positioned at the target? The authors suggest that the prior belief is informed by/related to motor noise. However, could the authors provide a bit more intuition about the sensory origin around this belief? Is it that, during the control task, participants build up an internal representation about the distribution of the visual cursor feedback signaling their actual hand position, and as such, the prior belief is informed by vision? Therefore, the ideal observer model is thus an integration between a memory of the visual distribution against a nosier proprioceptive distribution? Or, is the prior belief constructed by relying on a history of prospective proprioceptive cues (without vision) integrating with a flat prior on a trial-by-trial basis, and that this prior is constructed slowly by the distribution of estimated endpoints? That is, prior motor noise may be dependent/interact with proprioceptive noise. Or do the authors think that confidence judgments access 'motor noise', a signal that is not tied to any specific sensory origin?

Participants' prior beliefs about their hand location are based on the visual feedback trials in both the control and main tasks, plus a lifetime of reaching experience. Since the feedback is veridical (known to the observer) this provides a visual estimation of their motor error over multiple trials. Proprioception from a given reach can be compared to the visual feedback to allow participants to estimate the amount of error present in their sense of proprioception. We've added a sentence in the discussion to address where these prior assumptions arise from, p. 36 paragraph 3

3) Reliability of the data. The authors mention a control motor awareness task to estimate motor noise and proprioceptive noise. Would the authors be open to representing these data in the supplemental figure, especially a potential figure showing the reliability of motor/proprioceptive noise + fitting parameters on half of the data, correlated with fits on the other half? This seems like an important figure that would lend greater credence to the quality of the data in their main task.

We have added two figures depicting the data from the control task to the supplement. The first figure (p. 2 figure S2) shows the spread of reach endpoints for each participant around the target location, and the second figure (p. 3 figure S3) shows endpoints relative to reach endpoint.

In supplement section 4 we report the standard deviation as a percentage of ground-truth value across recovered parameters from 20 simulated data sets per base model, p. 6 paragraph 1

4) Validity of the confidence judgment. I think the confidence judgments are a cool continuous measure that moves us away from binary estimates of confidence (hit vs miss; side note: I did really like the following paper by the group that used binary judgments: (Gaffin-Cahn et al., 2019)). That being said, out of the 16 participants, only 8 show a significant correlation between confidence judgments and reach errors (Figure 17). Perhaps to complement this weak association, the authors could contrast this with a between-participants correlation of motor noise vs confidence judgments, to show that this confidence measure indeed tracks a behavioral measure.

As a broader indication that the confidence judgment is associated with motor performance we have added a figure to the supplement to show the significant correlation between average confidence for each participant and their motor noise, p. 9 figure S8.

The largely invariant confidence judgments within-participant may indeed reflect insensitivity to proprioception (for most individuals). But alternatively, it may simply reflect a strategy (risk aversion in setting a ring size that incorporates most motor noise), as the authors outlined in the Discussion. In addition, it may also reflect 'laziness' in their adjustments (setting the ring pretty much at a similar location), masquerading sensitivity to proprioceptive cues – this may reflect a response bias. To this end, it was not clear in the manuscript whether the authors jittered the initial size of the confidence ring before the participant's judgment to counterbalance out/discourage possible response biases. If so, it would be helpful to detail the starting position of the ring in the Methods section.

We are confident in asserting that this variation is not risk aversion, as the proportion of points earned is similar for both types of participants, point values noted on p. 29 paragraph 1.

The ring starting size was randomly generated at a size pulled uniformly from the range of radii that returned points (.1 to 7 cm radius). This has been clarified in the text, p. 15 paragraph 1.

Awesome work. I enjoyed reading the manuscript, thoroughly.

Thank you so much!!!

References

Gaffin-Cahn, E., Hudson, T. E., & Landy, M. S. (2019). Did I do that? Detecting a perturbation to visual feedback in a reaching task. Journal of Vision, 19(1), 5.

Ruttle, J. E., Hart, B. M. 't, & Henriques, D. Y. P. (2021). Implicit motor learning within three trials. Scientific Reports, 11(1), 1627.

Tsay, J. S., Parvin, D. E., & Ivry, R. B. (2020). Continuous reports of sensed hand position during sensorimotor adaptation. Journal of Neurophysiology, 124(4), 1122–1130.

Reviewer #2:

This work addresses the interesting question of what determines sensory-motor confidence. The authors hypothesize that people integrate multiple cues and design a task that affords continuous confidence measures and incentivizes both accurate performance and confidence judgments. They extend a Bayesian model of sensory-motor behavior to confidence judgments in this task. They fit competing models jointly to behavior on this task, as well as a control task, allowing them to constrain and estimate parameters that are otherwise not observable in the main task. They compare three models: An optimal Bayesian observer that integrates prospective information based on past observations, a retrospective model that only uses current, proprioceptive evidence, and a prospective model that only uses past experience. The authors find strong individual differences in which model captures behavior better, and that people with more reliable proprioception are more likely to integrate that information into their confidence judgments.

I found this study interesting, insightful, the experiment clever, the analyses rigorous, and the manuscript generally very clear. I have a minor comment and a question, but overall found this manuscript a pleasure to read.

Thank you so much!

One thing I found confusing is that in the figures for the different models the confidence circle was always the same size. That size should vary depending on where the estimated end-point is, thought, right? I think changing that would help clarify the different predictions these models make that allow them to be tested against each other. Perhaps visualizing that in one shared figure would also help tune people's intuitions.

As these figures are cartoons and not reflecting true data we erred on the side of readability and fitting everything within the confines of the figure. I have clarified in the figure 6, 7, 8 & 9 descriptions that these model figures are cartoons, p. 19-20, 23- 25.

Conceptually, I have a question about whether this procedure measures confidence per se versus error monitoring - that is without an uncertainty estimate associated with it, as in perfect endpoint estimation - and whether the authors think of these functions as distinct versus integrated. I understand that for continuous measures the conceptual space is still very muddy, so I don't expect the authors to necessarily address that in the paper - I am curious, though.

We have added a section in the discussion highlighting that this study focuses on the confidence one has in achieving their goal, and not the confidence they have in their true hand location. For example a participant could have high confidence in their true hand location and know for certain it is far away from the target, in our paradigm this would be reported as low confidence as they know there was very little chance they hit the target, p. 43 paragraph 1.

Reviewer #3: Review of "Feeling lucky? Prospective and retrospective cues for sensorimotor confidence"

This is an interesting and well-written paper combining experiment and modeling which studies the role of prospective and retrospective cues in a pointing task.

The main novelty here is the usage of a "interval-like" report (a circle) with a scoring system which would encourage subjects to report their uncertainty/confidence about their distance to the target on a continuous scale. Similar methods have been used extensively in perceptual studies of uncertainty/confidence (e.g., Honig, Ma & Fougnie, PNAS 2020), but I am not aware of this paradigm used in a sensorimotor study.

I think the paper is a very nice contribution to the sensorimotor modeling literature, with a few comments below. Thanks!

Major comments:

1. Model fit visualization:

Everything is well-executed and well-presented in terms of experimental design, computational modeling, model fitting, model comparison (with minor caveats), model recovery. However, the current submission does not show much about the model fits / posterior-predictive checks. In some sense, model comparison is meaningful only when the model set contains the true model (or, realistically, a close-enough model), otherwise it is just a model-fitting exercise (an exercise I am quite fond of, but still). In practice, the reader needs to be convinced that the best-fitting models are effectively capturing some key properties of the data, and this is done by showing the model fits various aspects of the data.

In this paper, as far as I can see, Fig 12 (and Fig 18 in the Appendix) are the only "fits" that we are shown. With a bit of a skeptical note, we could say that the distribution of "confidence circle size" in Fig 18 is for most subjects somewhere in the middle of the range, and the best-fitting model for each subject ends up simply being whichever model overlaps most with this distribution (sometimes it is one model, sometimes it is another model). Can the authors show some other summary statistics / observable of the data to indeed provide a stronger feel for how the models fit the data?

For example, instead of a distribution/histogram plot, the authors could show a scatter plot of true circle size vs. predicted circle size (for different models), so that at least we can see that there is a correlation between model predictions and actual responses on a trial-by-trial basis (which we cannot see from Fig 12/18), which would be a stronger evidence that the model is capturing a real process. Maybe there are also other plots that the authors can present to strengthen the model fit presentation.

We have added these scatter plots to the supplement, p. 11 figure S9

2. Alternative models?

As a follow-up to point 1: given the relatively little information about the model fits themselves - and part of the Discussion -, I am under the impression that no model fits the data particularly well here (but happy to be corrected by additional plots or analyses).

In particular, the reported model-switching between "ideal" and "prospective" model for different values of the proprioceptive uncertainty parameter is especially suggestive that there may be some other mechanism at play, which is not captured by any current model. The "switch" of the model, as mentioned previously, may just be accidental (i.e., the winning model is whichever happens to overlap with the circle distribution, which is dictated by the true underlying model).

Just to be clear, some bad fits (and a lack of a "true model") are common in psychophysics and we all agree it's fine as long as some important aspects of the data are captured by the presented models. However, I think in this case it's worth it for the authors to give a thought about alternative models, especially here since it seems that there is indeed some underlying pattern. I am not going to give specific advice since the goal is not to fit "that reviewer's favorite hypothesis", but just to think whether there might be other models to be considered. (There are plenty of ideas to be taken from the perceptual and decision-making domain.)

Of course, it's always a good thing to do model-comparison across a wide range of sensible models. Our research question was intentionally simple: do people use prior (prospective) information, trial-specific (retrospective) information or both in determining confidence in an action. We were concerned about the use of prior information in the control task and, in fact, we considered models of the control task using only prospective or only retrospective information (rather than the current models assume that both retrospective and prospective information are always used in the control task), but none of those fit better than those described in the paper, and so we chose to omit them from the manuscript. We're not sure what ideas you allude to "from the perceptual and decision-making domain." Sure, we could also throw in something about risk aversion or uncertainty aversion (warped reward and/or probability weights) from Prospect Theory, but we feel that would not add anything useful to the message of the current paper.

Minor comments:

- "For our model fits of behavioral data, the smallest BIC difference between the winning model and next-best model was 12.38, which is already strong evidence as guidelines suggest a score over 10 to be vigorously compelling (Raftery, 1995)."

I used to write this stuff as well (everybody does), but just a remark that this may be somewhat misleading. The statement above is correct when the true model is in the model set, but since this is never the case, its *interpretation* as "vigorously compelling" is debatable.

Updated the text to better classify this assumption, p. 35 paragraph 3

- First, differences of AIC/BIC in the hundreds are very common for a typical psychophysical experiment with hundreds of trials, but that's just a consequence of the concentration of probability. The fact that we are much more certain that a model is better than another does not mean that the winning model is anywhere close to the true model (I know the authors are not stating that, but the less experienced reader might be confused).

Clarified in the text that we are not implying that our model is for certain the true model, p. 35 paragraph 3

- Second, here we are "very certain" (i.e., large BIC difference) that some subjects are following the "ideal" model, and we are also "very certain" that some other subjects are following the "prospective" model, but this is also exactly what one would expect under model misspecification (i.e., lack of a true model). One possibility is that the true model is in some other parts of model space, and what happens here is that whichever model ends up slightly closer (for whatever reason) to the true model ends up winning by seemingly a large amount, but this is effectively just "noise" in model space.

Added a section in the discussion to speak to a potential misclassification when the true model is missing, p. 35-36 paragraph 3
