# Supplementary material for: Feeling lucky? Prospective and retrospective cues for sensorimotor confidence
Source: PLoS Comput Biol. 2023 Jun 26;19(6):e1010740. doi: 10.1371/journal.pcbi.1010740 (PMC10348589; doi:10.1371/journal.pcbi.1010740)
Supplement: Attachment — Submitted filename: Reviewer Response Round 2.pdf [file pcbi.1010740.s012.pdf]

Reviewer #3: Thanks to the authors for their careful revision and for addressing most of my concerns with changes in the paper.
Gladly!

However, my concern about the quality of the model fits is still there. The additional scatter plots (Fig S9) do not quite address my concerns. In Fig S9, there seems to be little correlation between the data and the model; the authors acknowledge that in the caption, but it doesn't change the fact that these model fits don't do much to showcase goodness of fit.
If adding simulated noise obfuscates whether the models are good or not, as I suggested in my previous review, the authors could come up with a relevant summary statistic (averaged over some trials or relevant conditions) that can be plotted over the model prediction (with error bars); a common practice to show an approximate match between data and model.

We have added three plots to the main text which compare elements of the behavior observed from the participants to those derived from the models. Specifically the proportion of trials on which a circle size was selected that successfully earned points, the total number of points earned during the experiment, and the average confidence report over all trials. As we noted previously, there is no way to know a participant's sensed location on a given trial. We simulate participants by sampling a sensed location from a distribution with SD equal to our best estimate of the participant's proprioceptive uncertainty. This results in a low trial-by-trial correlation between our simulated data and the behavioral data, because the sensed location determines the circle size for both the ideal and retrospective models. However, our models do capture participant-specific behavioral trends averaged over the experiment (see new Figure 13).

Overall, to better restate my main point: my only concern about this study is that the final analysis misses a critical assessment of whether the models truly capture features of the data. The model evaluation step is an essential part of the modeling workflow (e.g., see Gelman et al., 2020, "Bayesian workflow"). I encourage the authors to expand the model evaluation in this paper, e.g. producing some plots and analyses that support the validity of their models (some of which should be in the main text, and not relegated to the Appendix).

The new plots can be found on page 35 of the manuscript and within the text on page 33, highlighted in blue

For example, the authors could indeed show that their models capture relevant features of the data for multiple subjects (maybe some of the existing plots in the Appendix already do that; in which case it's enough to point the reader and highlight the relevant results). Conversely, the authors might come to acknowledge that the model fits are overall not great (as it is often the case in psychophysics!); but nonetheless they could argue that their study is still informative for this and that reason, which I would be also fine with.

To conclude, I think this is overall a good study, and I very much like the premise, the experiments and the modeling setup; I just feel the results (model fits, how they quantitatively and qualitatively relate to the data) and in particular the model evaluation part could be

expanded; and this would strengthen the paper by convincing the (nitpicky) reader of the validity of the results.

Thank you for the thoughtful comments and we hope you'll find the updated version to your satisfaction.
